# Proteomic analysis of an engineered isolate of *Lactobacillus plantarum* with enhanced raffinose metabolic capacity

**DOI:** 10.1038/srep31403

**Published:** 2016-08-11

**Authors:** Jicheng Wang, Wenyan Hui, Chenxia Cao, Rulin Jin, Caixia Ren, Heping Zhang, Wenyi Zhang

**Affiliations:** 1 Key Laboratory of Dairy Biotechnology and Engineering, Ministry of Education, Inner Mongolia Agricultural University, 306 Zhaowuda Road, Hohhot, Inner Mongolia 010018, China

## Abstract

Lactic acid bacteria that can produce alpha-galactosidase are a promising solution for improving the nutritional value of soy-derived products. For their commercial use in the manufacturing process, it is essential to understand the catabolic mechanisms that facilitate their growth and performance. In this study, we used comparative proteomic analysis to compare catabolism in an engineered isolate of *Lactobacillus plantarum* P-8 with enhanced raffinose metabolic capacity, with the parent (or wild-type) isolate from which it was derived. When growing on semi-defined medium with raffinose, a total of one hundred and twenty-five proteins were significantly up-regulated (>1.5 fold, *P* < 0.05) in the engineered isolate, whilst and one hundred and six proteins were significantly down-regulated (<−1.5 fold, *P* < 0.05). During the late stages of growth, the engineered isolate was able to utilise alternative carbohydrates such as sorbitol instead of raffinose to sustain cell division. To avoid acid damage the cell layer of the engineered isolate altered through a combination of *de novo* fatty acid biosynthesis and modification of existing lipid membrane phospholipid acyl chains. Interestingly, aspartate and glutamate metabolism was associated with this acid response. Higher intracellular aspartate and glutamate levels in the engineered isolate compared with the parent isolate were confirmed by further chemical analysis. Our study will underpin the future use of this engineered isolate in the manufacture of soymilk products.

Soy-derived products contain the alpha-galactooligosaccharide sugar, raffinose. Due to a lack of pancreatic alpha-galactosidase (α-Gal) that could catalyze its hydrolysis, humans are unable to digest this sugar^1^. However, this sugar is used by gas-producing bacteria in the large intestine, resulting in documented intestinal disorders such as nausea, diarrhoea and abdominal pain^2^. To overcome this drawback which reduces the nutritional value of soy products, much attention has been paid to the use of α-Gal-producing lactic acid bacteria (LAB) in the production of soy products. Isolates from several LAB species including *Lactobacillus curvatus*, *Lactobacillus plantarum*, *Lactobacillus acidophilus* and *Leuconostoc mesenteroides* have the potential to reduce raffinose levels in soy products^3^. Fermentation with these isolates could eliminate undesirable physiological effects associated with the consumption of soy products^4^.

In the last few years, modeling of the transport and catabolic pathways for raffinose have made significant progress alongside advances in the understanding of LAB genomics^5^. There have also been some studies that attempt to use genetic engineering techniques for the constructing isolates with enhanced α-Gal activity^6^. Within the genus *Lactobacillus*, the first study on the molecular mechanism behind raffinose utilization was done using microarrays, which revealed that genes involved in the metabolism of raffinose by *L. plantarum* WCSF were differentially expressed^7^. Recently, the genetic loci coding for the catabolic pathway of raffinose were accurately assigned in *L. acidophilus* NCFM, also using microarray techniques^8^. Both of these studies demonstrated the importance of understanding the catabolic pathway in selected strains if they are to be better exploited in industrialized production, but also the high efficiency of new genomic research tools.

*Lactobacillus plantarum* P-8 is a probiotic isolate from traditionally fermented dairy products^9^,^10^,^11^,^12^,^13^. It grows well in soymilk and is able to metabolize α-galactosides (stachyose and raffinose)^14^. In this study, comparative proteomic analysis was performed on an isolate of *L. plantarum* P-8 that had been engineered for enhanced raffinose metabolic capacity and its original parent strain. Our aim was to compare the behaviour of both isolates in the presence of raffinose. The information provided here will underpin the future use of probiotics with enhanced raffinose metabolism in the manufacturing of soymilk products.

## Results

### Growth of engineered *L. plantarum* P-8 and its parent isolate on media supplemented with raffinose

**Figure 1:**
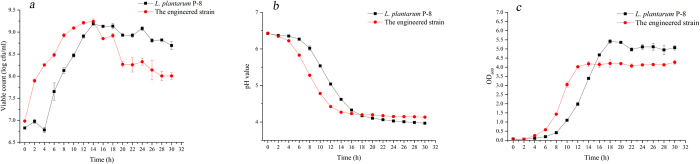
Growth curve of the engineered isolate of *L. plantarum* P-8 and its parent isolate in SDM supplemented with raffinose as determined by (**a**) viable counts; (**b**) pH; (**c**) OD values.

Growth curves based on viable counts, pH values and OD values were produced for both isolates growing in media supplemented with raffinose (Fig. 1a–c). Although the initial inoculum densities of the two strains in the media were the same, the growth rates were completely different. The number of viable counts increased more rapidly for the engineered isolate than the parent isolate although they ultimately achieved a similar density (above 9.24 × 10^7^ cfu/mL); thereafter the viable cell count fell off more rapidly in the engineered isolate than the parent isolate (Fig. 1a). Compared with its original strain, the pH of the semi-defined medium (SDM) inoculated with the engineered isolate dropped much faster than for the parent isolate indicating it more rapid fermentation rate (Fig. 1b).

### Organic acids after fermentation

**Figure 2:**
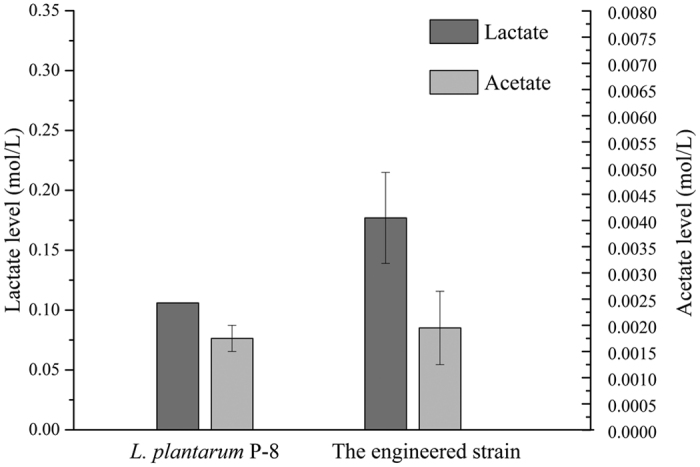
Concentration of lactate and acetate in SDM supplemented with raffinose following fermentation by an engineered isolate of *L. plantarum* P-8 or the parent isolate after 12 h cultivation.

After fermentation, lactic acid and acetate were the main end products present in the medium with raffinose (Fig. 2). A slightly higher concentration of these organic acids was detected in the medium fermented by the engineered isolate compared with the parent isolate (Fig. 2), which was in accordance with the pH values determined.

### Intracellular amino acid profile

A total of 17 intracellular amino acids were quantified in the engineered and parent isolates (Table 1). There were higher intracellular levels of aspartate and glutamate in the engineered isolate than in the parent isolate (Table 1). No serine or phenylalanine was detected within the growing cells.

### Up-regulation of proteins during late growth in media containing raffinose

**Figure 3:**
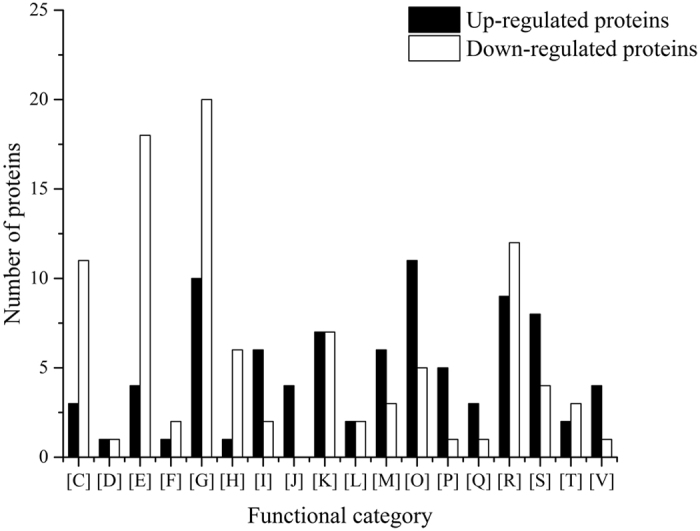
Clusters of orthologous groups of proteins (COGs) of differentially expressed proteins in the engineered isolate of *L. plantarum* P-8 compared with the parent isolate. Up-regulated proteins (black bars) and down-regulated proteins (white bars) are shown. Functional categories: [C], Energy production and conversion; [D], Cell cycle control, cell division, chromosome partitioning; [E], Amino acid transport and metabolism; [F] Nucleotide transport and metabolism; [G], Carbohydrate transport and metabolism; [H], Coenzyme transport and metabolism; [I], Lipid transport and metabolism; [J], Translation, ribosomal structure and biogenesis; [K], Transcription; [L], Replication, recombination and repair; [M], Cell wall/membrane/envelope biogenesis; [O], Posttranslational modification, protein turnover, chaperones; [P], Inorganic ion transport and metabolism; [Q], Secondary metabolites biosynthesis, transport and catabolism; [R], General function prediction only; [S], Function unknown; [T], Signal transduction mechanisms; [V], Defense mechanisms.

A total of 125 proteins were significantly up-regulated (>1.5 fold, *P* < 0.05) in the engineered isolate compared with the parent isolate (Table 2). Most of these proteins could be assigned to the category from the clusters of orthologous groups of proteins (COGs) (Fig. 3), with 12.6% proteins involved in posttranslational modification, protein turnover, chaperones and 11.5% of proteins involved in carbohydrate transport and metabolism.

Clustered genes (LBP_cg2912- LBP_cg2914, LBP_cg2917) could be distinguished that were significantly up-regulated (>3.8 fold). The set of sorbitol-related proteins consisted of a sorbitol PTS EIIA, a sorbitol PTS EIIBC, a sorbitol PTS EIIC and a sorbitol-6-phosphate 2-dehydrogenase. This was similar to the genetic organization of the sorbitol operon identified in *L. casei* ATCC BL23^15^. The operon coded in the parent isolate also included an activator.

Proteins associated with the cell membrane and cell wall metabolism, namely an alanine racemase (LBP_cg0413), a D-alanine-poly (phosphoribitol) ligase subunit (LBP_cg1580), a large-conductance mechanosensitive channel (LBP_cg1017), a glutamate racemase (LBP_cg1859) and a cyclopropane-fatty-acyl-phospholipid synthase (LBP_cg0412) were all up-regulated in the engineered isolate compared with the parent isolate. This may be because the rapid growth of the engineered isolate compared with the parent isolate means that it is likely to be challenged by a more acidic environment in the medium. Other characteristics of the acid response in the engineered isolate include activation of classic stress response proteins. These include a small heat shock protein (LBP_cg0109), a chaperone protein *dna*K (LBP_cg1586), a stress induced DNA binding protein (LBP_p6g011), an ATP-dependent *Clp* protease (LBP_cg2905), a 60 kDa chaperon and a cold shock protein *Csp*C (LBP_cg0785).

### Down-regulation of proteins during late growth on media containing raffinose

A total of 106 proteins were significantly down-regulated (<−1.5 fold, *P* < 0.05) in the engineered isolate compared with the parent isolate (Table 3). As can be seen from the COG category (Fig. 3), these proteins were mainly involved in amino acid transport and metabolism (18.2%), and carbohydrate transport and metabolism (20.2%).

Amongst them, proteins coding for an alpha-galactosidase (LBP_cg2832) and a beta-galactosidase (LBP_p2g004) have been predicted to be involved in the raffinose metabolism of LAB species^16^. Other repressed proteins associated with carbohydrate metabolism were involved in galactitol utilization (LBP_cg2877-LBP_cg2878) and the pentose phosphate pathway (LBP_cg2869 and LBP_cg2868). Two proteins coding for an aspartate aminotransferase (LBP_p3g040) and an AAE family aspartate:alanine exchanger (LBP_p3g041) were found to be flanked by transposases in the plasmid. Unexpectedly, some transporters of oligopeptides (LBP_cg0966 and LBP_cg0963) and amino acids (LBP_cg0653, LBP_cg0601 and LBP_cg0602) were detected, suggesting a low requirement for these materials during the late growth of the engineered isolate.

## Discussion

LAB isolates that can produce α-Gal are a promising way to improve the nutritional value of soy-derived products. For their further exploitation in the manufacturing process, it is important to understand the catabolism mechanisms involved in growth. In the present study we used comparative proteomic analysis to compare the metabolic capacity of an engineered isolate of *L. plantarum* with the parent isolate when growing in media supplemented with raffinose.

Isolates of *L. plantarum* are included on the list of α-Gal producing LAB^17^. Genes coding for α-Gal hydrolysis have been characterized at the biochemical and molecular levels in *L. plantarum*^18^. In the isolate *L. plantarum* ATCC 8014, genes involved in galactoside catabolism were clustered in a galactose operon^16^. The protein coding for α-Gal that often initiates the first degradation step in α-Gal hydrolysis of α-1,6-galactoside links in raffinose were flanked by a raffinose transporter and two subunits of the heteromdimeric β-galactosidase^16^. Inspection of the genome of the isolate used in this study (*L. plantarum* P-8) revealed a cluster of genes with the same organization on the chromosome^12^. In addition, a copy of the cluster, except for the raffinose transporter, was found on a plasmid^12^. Redundant coding genes associated with α-Gal in *L. plantarum* P-8 seem to endow this isolate with a good performance in the presence of the soy-derived products^14^. In the present study, some of these proteins were down-regulated in the engineered isolate, consistent with the fact that most of the raffinose was depleted from the medium by the late stage of its growth. In contrast the parent isolate, which had a slower growth rate still required raffinose as the sole carbohydrate source to support its growth at the same stage.

Sorbitol, also referred to as D-glucitol, is unlikely to have been present in the medium used in our study, but could be produced at a low level as the by-product of *L. plantarum* fermentation^19^. Within the sorbitol-related protein set, sorbitol-6-phosphate 2-dehydrogenase (*Srl*D) that usually catalyzes the conversion of sorbitol-6-phosphate to fructose-6-phosphate^20^, was the most highly expressed. Similarly, Laakso *et al.*^21^ found that the expression of *Srl*D and glucitol/sorbitol-PTS increased over time in *L. rhamnosus* GG during growth in industrial-type whey medium, especially when the culture shifted from the exponential growth phase to the stationary phase. They therefore proposed that *L. rhamnosus* GG began to use alternative energy sources, namely sorbitol, at the beginning of the stationary phase. This also seems to be a reasonable interpretation of the up-regulation of proteins for sorbiol utilization observed in our study, because the engineered isolate was entering the stationary phase at the time of sampling (Fig. 1).

Acid stress in LAB often invokes a variety of protection mechanisms^22^. Amongst them, the structure of cell layers is considered to be a significant factor in sensing the acidic environment^23^. Alteration of the cell layer by changing the saturated and cyclopropane fatty acids (FA) of the membrane in response to acidification has been observed in *L. casei* ATCC 334^24^. The authors suggested that increasing the rigidity and compactness of the cytoplasmic membrane decreased the permeability for protons^24^. For the engineered isolate we showed that the alteration of the cell layer occurred through a combination of *de novo* FA biosynthesis and modification of existing lipid membrane phospholipid acyl chains. In addition to the proteins related to cell wall metabolism, a cyclopropane-fatty-acyl-phospholipid synthase was up-regulated, as well as a protein that catalyzes the reactions for modifying the lipid membrane. Interestingly, glutamate racemase, which is involved in constructing cell walls^25^, was also induced, suggesting high levels of intracellular glutamate. Consistently, higher intracellular glutamate levels were found in the medium in which the engineered isolate had been growing compared with the parent isolate.

Another interesting finding was the regulation of aspartate metabolism in the engineered isolate. Two proteins involved in aspartate metabolism were significantly depressed. According to Wu *et al.*^26^, the twist of aspartate flux may help *L. casei* to perform better under acid stress. Similarly, we observed that the engineered isolate had a greater capacity to manipulate aspartate metabolism by enriching higher amounts of the intracellular aspartate.

In this study, comparative proteomic analysis was used to compare the metabolic capacity of an engineered isolate of *L. plantarum* with its parent isolate. During the late stages of growth, the engineered isolate used alternative carbohydrate such as sorbitol instead of raffinose to sustain its cell division. To avoid acid damage the engineered isolate altered the cell layer through a combination of *de novo* FA biosynthesis and modification of existing lipid membrane phospholipid acyl chains. Interestingly, aspartate and glutamate metabolism was associated with this acid response. Our study contributes to underpinning the future use of these isolates in manufacturing soy-milk products.

## Methods

### Bacterial isolates and culture conditions

An engineered isolate of *L. plantarum* P-8 with enhanced raffinose metabolic capacity and its original parent isolate were cultured in SDM supplemented with 1.0% (w/w) raffinose. The engineered isolate was obtained from a laboratory evolution experiment that lasted for 150 days. During the experimental process, *L. plantarum* P-8 was continuously subcultured in de Man-Rogosa-Sharpe broth for lactobacilli with 0.2 g/L glucose (unpublished data). The composition of the SDM was as described by Kimmel *et al.*^27^. A growth curve was constructed in relation to optical density (OD), pH and the number of viable counts determined after 0, 2, 4, 6, 8, 10, 12 14, 16, 18, 20, 22, 24, 26, 28, 30 and 32 h of fermentation. All analyses were performed in triplicate.

### Sample preparation

To ensure the reliability of the proteomic analysis, samples were obtained from 4 biological replicates after 12 h cultivation. Cells of the two isolates were harvested by centrifugation and washed with phosphate buffered saline (PBS) four times. One milliliter of lysis buffer (7 M urea, 4% sodium dodecyl sulfate, 30 mM 4-(2-hydroxyerhyl) piperazine-1-erhaesulfonic acid, 1 mM phenylmethylsulfonyl fluoride, 2 mM ethylenediamine tetraacetic acid, 10 mM DL-dithiothreitol, 1× protease inhibitor cocktail) was added to each sample, followed by sonication on ice and centrifugation at 13, 000 rpm for 10 min at 4 °C. The supernatants from each sample were transferred to fresh tubes.

### Protein digestion and isobaric tags for relative or absolute quantitation (iTRAQ) labeling

We determined the protein concentration of the supernatants using the bicinchoninic acid protein assay, and then transferred 100 μg protein per condition into new tubes and adjusted each to a final volume of 10 μL with 100 mM triethylammonium bicarbonate (TEAB). To this 5 μL of 200 mM DL-dithiothreitol were added and incubated at 55 °C for 1 h, then 5 μL of the 375 mM iodoacetamide was added to the sample and incubated for 30 min protected from light at room temperature.

For each sample, proteins were precipitated with ice-cold acetone, and then re-dissolved in 20 μL TEAB. Proteins were then digested with sequence-grade modified trypsin (Promega, Madison, WI), and the resultant peptide mixture was labeled using chemicals from the iTRAQ reagents kit (Applied Biosystems, Foster City, CA). The labeled samples were combined, desalted using a C18 SPE column (Sep-Pak C18, Waters, Milford, MA) and dried under vacuum.

### High pH reverse phase separation

Phase separation was performed as described by Gilar^28^ with some modifications. The peptide mixture was dissolved in buffer A (buffer A: 10 mM ammonium formate in water, pH10.0, adjusted with ammonium hydroxide), and then fractionated by high pH separation using an Aquity UPLC system (Waters Corporation, Milford, MA) connected to a reverse phase column (BEH C18 column, 2.1 mm × 150 mm, 1.7 μm, 300 Å, Waters Corporation, Milford, MA). High pH separation was done using a linear gradient. Starting from 0% B to 45% B in 35 min (B: 10 mM ammonium formate in 90% acetonitrile, pH 10.0, adjusted with ammonium hydroxide). The column flow rate was maintained at 250 μL/min and column temperature was maintained at 45 °C. Sixteen fractions were collected and each fraction was dried in a vacuum concentrator prior to the next step.

### Low pH nanoscale high-performance liquid chromatography coupled to tandem mass spectrometry (nano-HPLC-MS/MS) analysis

The fractions were re-suspended in a mixture of solvent C and solvent D (C: water with 0.1% formic acid; D: acetonitrile with 0.1% formic acid), separated by nano LC and analyzed by on-line electrospray tandem mass spectrometry. The experiments were performed on a Nano Aquity UPLC system (Waters Corporation, Milford, MA) connected to a quadrupole-orbitrap mass spectrometer (Q-Exactive) (Thermo Fisher Scientific, Bremen, Germany) equipped with an online nano-electrospray ion source. 8 μl peptide sample was loaded onto the trap column (Thermo Scientific Acclaim PepMap C18, 100 μm × 2 cm), with a flow of 10 μl/min for 3 min and subsequently separated on the analytical column (Acclaim PepMap C18, 75 μm × 25 cm) with a linear gradient, from 5% D to 30% D in 95 min. The column was re-equilibrated at initial conditions for 15 min. The column flow rate was maintained at 300 nL/min and column temperature was maintained at 45 °C. An electrospray voltage of 2.0 kV was used against the inlet of the mass spectrometer.

The Q-Exactive mass spectrometer was operated in the data-dependent mode to switch automatically between MS and MS/MS acquisition. Survey full-scan MS spectra (m/z 350-1600) were acquired with a mass resolution of 70 K, followed by fifteen sequential high-energy-collisional-dissociation (HCD) MS/MS scans with a resolution of 17.5 K. In all cases, one micro-scan was recorded using dynamic exclusion of 30 seconds. MS/MS fixed first mass was set at 100.

### Database searching

Tandem mass spectra were extracted by Proteome Discoverer software (Thermo Fisher Scientific, version 1.4.0.288). Charge state deconvolution and deisotoping were not performed. All MS/MS samples were analyzed using Mascot (Matrix Science, London, UK; version 2.3). Mascot was set up to search the NCBI database (Taxonomy: *Lactobacillus plantarum* P-8, 3179 entries) assuming the digestion enzyme trypsin. Mascot was searched with a fragment ion mass tolerance of 0.050 Da and a parent ion tolerance of 10.0 PPM. Carbamidomethylation of cysteine and iTRAQ 8plex of lysine and the n-terminus were specified in Mascot as fixed modifications. Oxidation of methionine and iTRAQ 8plex of tyrosine were specified in Mascot as a variable modification.

### Quantitative data analysis

We used the percolator algorithm lower than 1% to control peptide level false discovery rates (FDR). Only unique peptides were used for protein quantification and the method of normalization on protein median was used to correct experimental bias. The minimum number of proteins that must be observed was set to 1000. Statistical analysis was realized in the software package R; using students’t tests, *p* < 0.05 was considered statistically significant. A 1.5-fold change was used as the threshold for selection of regulated proteins. All regulated proteins were distributed over COGs and were subjected to the Kyoto Encyclopedia of Genes and Genomes (KEGG) database^29^.

### Measurement of organic acids

The content of lactate and acetate was determined by HPLC using the methods of Wang *et al.*^30^. Firstly, 1 mol/L HCl was used to denature protein at a volume of four times that of the samples. Then the samples were subjected to high speed centrifugation at 4, 200 rpm for 10 min. The supernatants were used for analysis after filter sterilization through a 0.45 μm filter. The mobile phase consisted of a phosphate buffered solution and methanol (97/3, v/v), with a flow rate of 0.5 mL/min. The UV detector was set at 210 nm and the ZORBAX SB-Aq column (5 μm, 4.6 × 150 mm, Agilent, USA) was operated at 35 °C.

### Quantification of intracellular amino acids

Extraction of intracellular amino acids was achieved as described by Wu *et al.*^26^. The amino acids were quantified using a Hitachi L-8900 fully automatic amino acid analyzer (Hitachi High-Technologies Corporation, Tokyo, Japan), which used ion-exchange chromatography to separate amino acids^31^.

## Additional Information

**How to cite this article**: Wang, J. *et al.* Proteomic analysis of an engineered isolate of *Lactobacillus plantarum* with enhanced raffinose metabolic capacity. *Sci. Rep.* **6**, 31403; doi: 10.1038/srep31403 (2016).

## Figures and Tables

**Table 1:** Concentration of intracellular amino acids in an engineered isolate of *L. plantarum* P-8 and its parent isolate after fermentation in SDM for 12 h.

Amino acid	Content (mg/L)	
	*L. plantarum* P-8	The engineered strain
Aspartic acid	0.151 ± 0.011^b^	0.422 ± 0.054^a^
Threonine	0.152 ± 0.007^a^	0.141 ± 0.037^a^
Serine	0.049 ± 0.005^a^	0.031 ± 0.006^b^
Glutamine	1.061 ± 0.073^b^	1.585 ± 0.225^a^
Glycine	0.092 ± 0.004^b^	0.114 ± 0.012^a^
Alanine	7.489 ± 0.393^a^	4.952 ± 0.285^b^
Cysteine	0.492 ± 0.010^a^	0.470 ± 0.006^b^
Valine	0.395 ± 0.016^b^	0.594 ± 0.020^a^
Methionine	0.429 ± 0.040^a^	0.498 ± 0.061^a^
Isoleucine	0.110 ± 0.005^a^	0.084 ± 0.010^b^
Leucine	0.154 ± 0.014^a^	0.173 ± 0.025^a^
Tyrosine	0.979 ± 0.019^a^	0.819 ± 0.336^a^
Phenylalanine	0.291 ± 0.011	—
Lysine	0.731 ± 0.042^a^	0.682 ± 0.059^a^
Histidine	0.167 ± 0.009^a^	0.133 ± 0.016^b^
Arginine	0.301 ± 0.031^a^	0.264 ± 0.024^a^
Proline	0.246 ± 0.023^a^	0.114 ± 0.009^b^

^a,b^Within the same row followed by different superscript letters differ significantly (*P* < 0.05).

**Table 2:** Up-regulated proteins in the engineered isolate of *L. plantarum* P-8 compared with its parent isolate.

Function group and ORF	Description	Fold change
Energy production and conversion		
LBP_cg2666	Glutathione reductase	1.56
LBP_cg1616	D-lactate dehydrogenase	1.79
Cell cycle control, cell division, chromosome partitioning		
LBP_cg1932	Bacterial cell division membrane protein FtsW	1.86
Amino acid transport and metabolism		
LBP_cg0323	Glycine betaine/carnitine/choline ABC transporter, substrate binding and permease protein	1.65
LBP_cg1441	putative 5-methyltetrahydropteroyltriglutamate–homocysteine S-methyltransferase	1.5
LBP_cg1849	Xaa-Pro dipeptidase	1.54
LBP_cg2294	Amino acid transport protein	1.69
Carbohydrate transport and metabolism		
LBP_cg1118	Cell-cycle regulation histidine triad protein	1.52
LBP_cg0026	Maltose phosphorylase	1.81
LBP_cg0226	Alpha, alpha-phosphotrehalase	1.54
LBP_cg0227	Phosphoenolpyruvate-dependent sugar PTS family porter EIIABC, trhalose specific	1.85
LBP_cg0470	Mannose PTS, EIIA	1.51
LBP_cg2634	Phosphotransferase system fructose-specific component IIA	1.59
LBP_cg2912	Sorbitol PTS, EIIA	5.8
LBP_cg2913	Sorbitol PTS, EIIBC	5.72
LBP_cg2914	Sorbitol PTS, EIIC	3.81
Lipid transport and metabolism		
LBP_cg0412	Holo-[acyl-carrier-protein] synthase	2.82
LBP_cg0649	Acyltransferase (Putative)	1.54
LBP_cg2725	Phosphatidylglycerophosphatase	2.01
LBP_cg1580	D-alanine–poly(phosphoribitol) ligase subunit 2-1	2.09
LBP_cg2490	Short-chain dehydrogenase/oxidoreductase	1.95
LBP_cg2917	Sorbitol-6-phosphate 2-dehydrogenase	10.6
Translation, ribosomal structure and biogenesis		
LBP_cg0495	50S ribosomal protein L33	3.28
LBP_cg0799	Seryl-tRNA synthetase	1.56
LBP_cg1733	30S ribosomal protein S20	1.51
LBP_cg1826	Polyribonucleotide nucleotidyltransferase (Putative)	1.58
Transcription		
LBP_cg0225	GntR family transcriptional regulator	2.17
LBP_cg0785	Cold shock protein CspC	1.72
LBP_cg1077	Arginine regulator	1.69
LBP_cg1588	Heat-inducible transcription repressor hrcA	2.06
LBP_cg2038	Hypothetical protein	1.59
LBP_cg2611	Transcription regulator	6.92
LBP_cg1703	Transcription regulator of fructose operon	1.65
LBP_cg2916	Sorbitol operon transcription regulator	1.78
Replication, recombination and repair		
LBP_cg1218	Exodeoxyribonuclease 7 small subunit	1.96
LBP_cg1718	UvrABC system protein C	1.5
Cell wall/membrane/envelope biogenesis		
LBP_cg0413	Alanine racemase	2.73
LBP_cg0619	Glucosamine–fructose-6-phosphate aminotransferase	2.15
LBP_cg1017	Large-conductance mechanosensitive channel	1.67
LBP_cg1636	Prophage Lp1 protein 58, lysin	2.21
LBP_cg1859	Glutamate racemase	1.9
LBP_cg2578	Cyclopropane-fatty-acyl-phospholipid synthase	1.64
LBP_cg2781	Extracellular protein, gamma-D-glutamate-meso-diaminopimelate muropeptidase (Putative)	2.83
Posttranslational modification, protein turnover, chaperones		
LBP_cg0109	Small heat shock protein	3
LBP_cg0196	Glutathione peroxidase	2.04
LBP_cg0537	10 kDa chaperonin	2.24
LBP_cg0538	60 kDa chaperonin	1.81
LBP_cg0971	ATP-dependent Clp protease, ATP-binding subunit ClpE	1.52
LBP_cg1585	Chaperone protein dnaJ	1.52
LBP_cg1586	Chaperone protein dnaK	2.11
LBP_cg1587	Protein grpE	1.73
LBP_cg2734	Small heat shock protein	1.87
LBP_cg2905	ATP-dependent Clp protease, ATP-binding subunit ClpL	1.85
LBP_cg2160	Thioredoxin H-type	1.68
Inorganic ion transport and metabolism		
LBP_cg0542	Phosphate ABC transporter, substrate binding protein	1.94
LBP_cg0659	Metal uptake regulator	1.75
LBP_cg2648	Ferric uptake regulator	2.09
LBP_cg2900	Catalase	1.71
LBP_p6g011	Stress induced DNA binding protein	1.92
General function prediction only		
LBP_cg0025	Beta-phosphoglucomutase	1.54
LBP_cg0197	Oxidoreductase	1.51
LBP_cg0344	Cyanide hydratase	2.18
LBP_cg2033	Prophage Lp3 protein 8, helicase	2.22
LBP_cg2366	NADP oxidoreductase coenzyme F420-dependent	1.63
Signal transduction mechanisms		
LBP_cg1346	Putative universal stress protein	1.5
LBP_p7g009	PemI-like protein	1.66
Defense mechanisms		
LBP_cg2242	ABC transporter, ATP-binding protein	1.77
LBP_cg2243	ABC transporter, permease protein	1.71
LBP_cg2310	ABC transporter, permease protein (Putative)	1.53
LBP_cg2311	ABC transporter, ATP-binding protein	1.83
Function unknown		
LBP_cg0151	Maltose/maltodextrin ABC transporter subunit (Putative)	1.65
LBP_cg0721	Alkaline shock protein	2.43
LBP_cg0722	Alkaline shock protein	2.92
LBP_cg1404	Hypothetical protein	1.64
LBP_cg1866	Hypothetical protein	1.54
LBP_cg1913	Hypothetical protein	1.64
LBP_cg2375	Integral membrane protein	2.6
LBP_cg2385	Integral membrane protein	1.65
LBP_cg0392	Hypothetical protein	1.79
LBP_cg0481	Extracellular zinc metalloproteinase	6.94
LBP_cg0600	Cell surface protein	2.45
LBP_cg0625	Hypothetical protein	1.53
LBP_cg0719	Hypothetical protein	1.92
LBP_cg0720	Hypothetical protein	3.17
LBP_cg0889	Hypothetical protein	2.66
LBP_cg1033	Extracellular protein, membrane-anchored (Putative)	2.44
LBP_cg1196	Hypothetical protein	10.39
LBP_cg1257	Cell surface protein	1.85
LBP_cg1314	Hypothetical protein	4.2
LBP_cg1367	Lysin	1.69
LBP_cg1637	Prophage Lp2 protein 54	1.77
LBP_cg1638	Prophage Lp2 protein 53	3.22
LBP_cg1639	Hypothetical protein	1.51
LBP_cg1640	Hypothetical protein	2.61
LBP_cg1641	Tail fiber	4.79
LBP_cg1642	Hypothetical protein	2.02
LBP_cg1643	Hypothetical protein	1.52
LBP_cg1646	Hypothetical protein	2.08
LBP_cg1647	Phage major tail protein	2.12
LBP_cg1651	Phage protein DNA packaging protein	2.25
LBP_cg1661	RinA family phage transcriptional regulator	2.15
LBP_cg1663	Hypothetical protein	3
LBP_cg1664	Hypothetical protein	4.27
LBP_cg1673	Hypothetical protein	2.16
LBP_cg1674	Hel protein	1.62
LBP_cg1675	Hypothetical protein	2.72
LBP_cg1677	Hypothetical protein	2.02
LBP_cg1682	Hypothetical protein	3.01
LBP_cg1721	Hypothetical protein	7.13
LBP_cg1851	Hypothetical protein	1.55
LBP_cg1933	Hypothetical protein	1.54
LBP_cg1962	Hypothetical protein	1.6
LBP_cg1970	Hypothetical protein	1.82
LBP_cg2009	Hypothetical protein	1.6
LBP_cg2032	Prophage Lp4 protein 12	1.64
LBP_cg2034	Prophage Lp3 protein 7	1.56
LBP_cg2185	Hypothetical protein	8.34
LBP_cg2289	Cell surface protein	2.84
LBP_cg2450	Hypothetical protein	15.41
LBP_cg2519	Extracellular protein (Putative)	3.74
LBP_cg2530	Muramidase (Putative)	1.84
LBP_cg2736	Hypothetical protein	30.12
LBP_cg2777	Transcription regulator	1.5
LBP_cg2779	Hypothetical protein	3.75
LBP_cg2898	Hypothetical protein	2.43

**Table 3:** Down-regulated proteins in the engineered isolate of *L. plantarum* P-8 compared with its parent isolate.

Function group and ORF	Description	Fold change
Energy production and conversion		
LBP_p2g050	Pyridine nucleotide-disulfide oxidoreductase family protein	−10.15
LBP_cg2288	Flavodoxin	−4.62
LBP_cg2434	Nitroreductase	−3.34
LBP_cg0327	Glycerol-3-phosphate dehydrogenase	−3.15
LBP_cg0326	Glycerol kinase 1	−2.67
LBP_cg2703	Formate C-acetyltransferase	−2.19
LBP_cg0631	Glycerol kinase 2	−2.09
LBP_cg2927	Bifunctional acetaldehyde-CoA/alcohol dehydrogenase	−2.03
LBP_cg0052	Nitroreductase	−1.93
LBP_cg0871	L-lactate dehydrogenase 2	−1.71
LBP_cg0092	Oxidoreductase	−1.76
Cell cycle control, cell division, chromosome partitioning		
LBP_p1g033	Copy number control protein	−18.2
Amino acid transport and metabolism		
LBP_cg0219	Cystathionine beta-lyase	−36.76
LBP_p3g040	Aspartate aminotransferase	−18.9
LBP_cg0220	Cysteine synthase	−6.96
LBP_cg0585	Lipoprotein, peptide binding protein OppA-like protein	−4.87
LBP_cg0963	Oligopeptide ABC superfamily ATP binding cassette transporter, substrate binding protein	−3.86
LBP_cg0017	Lipoprotein, peptide binding protein OppA-like protein	−3.55
LBP_p7g004	ABC-type polar amino acid transport system, ATPase component	−2.78
LBP_cg0602	Glutamine ABC transporter, ATP-binding protein	−2.45
LBP_cg0601	Glutamine ABC transporter, substrate binding and permease protein	−2.32
LBP_cg2273	Putative D-serine dehydratase	−1.96
LBP_cg2253	Bifunctional cystathionine gamma-lyase/maltose regulon repressor	−1.55
LBP_cg0653	Amino acid transport protein (Putative)	−1.5
LBP_cg2156	Pyruvate oxidase	−3.17
LBP_cg2911	Pyruvate oxidase	−1.85
LBP_cg0966	Oligopeptide ABC transporter, ATP-binding protein	−2.01
LBP_cg2875	L-iditol 2-dehydrogenase	−1.75
LBP_p7g005	ABC-type amino acid transport/signal transduction system, periplasmic component/domain protein	−3.67
LBP_cg0650	X-prolyl-dipeptidyl aminopeptidase	−1.59
Nucleotide transport and metabolism		
LBP_cg2224	Phosphoribosylamine–glycine ligase	−2.34
LBP_cg0658	Guanylate kinase	−1.59
Carbohydrate transport and metabolism		
LBP_p1g007	Cupin 2 conserved barrel domain protein	−20.36
LBP_p2g004	Beta-galactosidase	−19.36
LBP_p2g010	GPH family glycoside-pentoside-hexuronide:cation symporter	−18.77
LBP_p2g005	Beta-galactosidase large subunit	−15.86
LBP_cg2869	Putative transaldolase	−2.8
LBP_cg2868	Transketolase	−2.2
LBP_cg1489	Pyruvate,water dikinase	−2.07
LBP_cg1967	Prophage Lp2 protein 59; xylanase/chitin deacetylase (Putative)	−1.98
LBP_cg1383	Phosphoglycerate mutase (Putative)	−1.87
LBP_cg0206	Protein-N(Pi)-phosphohistidine–sugar phosphotransferase	−1.81
LBP_cg2857	Beta-glucosides PTS, EIIBCA	−1.73
LBP_cg2862	Alpha-glucosidase	−1.66
LBP_cg0954	6-phosphogluconate dehydrogenase, decarboxylating	−1.62
LBP_cg0148	Maltose/maltodextrin ABC transporter, substrate binding protein	−1.6
LBP_cg2856	6-phospho-beta-glucosidase	−1.58
LBP_cg2832	Alpha-galactosidase	−1.57
LBP_cg0146	Alpha-glucosidase	−1.56
LBP_cg2877	Galactitol PTS, EIIB	−1.55
LBP_cg0767	Alpha-ribazole-5′-phosphate phosphatase (Putative)	−1.51
LBP_cg2878	Galacitol PTS, EIIA	−1.59
Coenzyme transport and metabolism		
LBP_cg2237	Lipoate-protein ligase	−2.32
LBP_cg1190	5-formyltetrahydrofolate cyclo-ligase	−1.97
LBP_cg0655	Pyridoxal kinase	−1.81
LBP_cg1219	Geranyltranstransferase	−1.59
Lipid transport and metabolism		
LBP_cg1286	(3R)-hydroxymyristoyl-(Acyl carrier protein) dehydratase	−2.05
LBP_cg2422	Putative acyltransferase	−1.64
Transcription		
LBP_cg2245	Transcription regulator	−1.71
LBP_cg2858	Transcription antiterminator	−1.66
LBP_cg2353	Transcription regulator	−1.58
LBP_cg2921	Ribose operon repressor	−1.51
LBP_cg0477	Transcription regulator	−1.51
LBP_cg2612	Putative aromatic-amino-acid transaminase	−6.1
LBP_cg1416	Transcription repressor	−2.06
Replication, recombination and repair		
LBP_p1g016	Resolvase	−3.34
LBP_cg2331	Methylated-DNA-(Protein)-cysteine S-methyltransferase	−2.41
Cell wall/membrane/envelope biogenesis		
LBP_cg1351	Penicillin binding protein 1A	−3.18
LBP_cg1937	UDP-N-acetylglucosamine 1-carboxyvinyltransferase 1	−2.05
LBP_cg1793	Penicillin binding protein 2B	−1.78
Posttranslational modification, protein turnover, chaperones		
LBP_p2g013	Cell envelope-associated proteinase, lactocepin PrtR	−5.22
LBP_cg2704	Formate acetyltransferase activating enzyme	−2.23
LBP_cg1561	Protein-methionine-S-oxide reductase	−1.78
LBP_cg0698	Hypothetical protein	−1.67
LBP_cg1426	Peptide methionine sulfoxide reductase msrB	−1.52
LBP_p1g026	Multicopper oxidase	−1.61
General function prediction only		
LBP_p3g041	AAE family aspartate:alanine exchanger	−12.55
LBP_cg2520	HAD superfamily hydrolase	−2.77
LBP_cg2383	ABC superfamily ATP binding cassette transporter, ABC protein	−2.36
LBP_cg1029	HAD superfamily hydrolase	−2.28
LBP_cg2267	Acetyltransferase	−1.69
LBP_cg1295	GTPase	−1.67
LBP_cg2080	ABC superfamily ATP binding cassette transporter, ABC protein	−1.63
LBP_cg0694	2-nitropropane dioxygenase	−1.62
LBP_cg2803	NADH oxidase	−1.52
Signal transduction mechanisms		
LBP_p1g017	Putative universal stress protein	−5.94
Defense mechanisms		
LBP_cg1584	Serine-type D-Ala-D-Ala carboxypeptidase	−1.57
Function unknown		
LBP_p2g025	Hypothetical protein	−7.13
LBP_cg0505	Lysyl-tRNA synthetase (Class II)	−2.2
LBP_cg1165	Lipoprotein	−2.13
LBP_cg0676	Hypothetical protein	−1.83
LBP_p3g034	Hypothetical protein	−10.71
LBP_p3g025	Hypothetical protein	−6.26
LBP_p2g017	Nisin resistance protein	−6.01
LBP_cg1057	Hypothetical protein	−3.6
LBP_p3g035	LtrC-like protein	−3.59
LBP_cg2528	NmrA family protein	−3.24
LBP_cg0079	Hypothetical protein	−2.4
LBP_cg1398	Hypothetical protein	−2.18
LBP_cg1623	Hypothetical protein	−2.1
LBP_cg1376	Pore-forming protein	−2.09
LBP_p3g036	Hypothetical protein	−1.83
LBP_cg2330	Extracellular protein	−1.76
LBP_cg0725	Lipoprotein	−1.73
LBP_cg2475	Extracellular protein	−1.6
LBP_cg1507	Hypothetical protein	−1.56
